# Development and validation of a prognostic model for kidney renal clear cell carcinoma based on RNA binding protein expression

**DOI:** 10.18632/aging.104137

**Published:** 2020-11-20

**Authors:** Yuzhu Xiang, Shengcai Zhou, Jian Hao, Chunhong Zhong, Qimei Ma, Zhuolun Sun, Chunxiao Wei

**Affiliations:** 1 Department of Urology, Shandong Provincial Hospital Affiliated to Shandong First Medical University, Jinan 250021, Shandong, China; 2 Department of Urology, Yiyuan County People's Hospital, Zibo 256100, Shandong, China; 3 Department of Urology, Xintai People's Hospital, Xintai 271200, Shandong, China; 4 Department of Central Sterile Supply, Shandong Provincial Hospital Affiliated to Shandong First Medical University, Jinan 250021, Shandong, China; 5 Department of Rehabilitation Medicine, Shandong Provincial Hospital Affiliated to Shandong First Medical University, Jinan 250021, Shandong, China; 6 Department of Urology, The Third Affiliated Hospital of Sun Yat-sen University, Guangzhou 510630, China

**Keywords:** kidney renal clear cell carcinoma, nomogram, prognostic model, RNA binding protein, The Cancer Genome Atlas

## Abstract

Dysregulated expression of RNA-binding proteins (RBPs) is strongly associated with the development and progression of multiple tumors. However, little is known about the role of RBPs in kidney renal clear cell carcinoma (KIRC). In this study, we examined RBP expression profiles using The Cancer Genome Atlas database and identified 133 RBPs that were differentially expressed in KIRC and non-tumor tissues. We then systematically analyzed the potential biological functions of these RBPs and established PPIs. Based on Lasso regression and Cox survival analyses, we constructed a risk model that could independently and accurately predict prognosis based on seven RBPs (NOL12, PABPC1L, RNASE2, RPL22L1, RBM47, OASL, and YBX3). Survival times were shorter in patients with high risk scores for cohorts stratified by different characteristics. Gene set enrichment analysis was also performed to further understand functional differences between high- and low-risk groups. Finally, we developed a clinical nomogram with a concordance index of 0.792 for estimating 3- and 5-year survival probabilities. Our results demonstrate that this risk model could potentially improve individualized diagnostic and therapeutic strategies.

## INTRODUCTION

Renal cell carcinoma (RCC), one of the most common urinary system tumors, accounts for approximately 3% of all adult cancers and is responsible for more than 400,000 new diagnoses and 140,000 cancer-related deaths annually worldwide [1]. Kidney renal clear cell carcinoma (KIRC), the most common pathological subtype of RCC, is associated with high mortality rates and poor prognosis because of its aggressive growth and high metastasis rates [2]. Because KIRC patients usually not do not respond to traditional radiotherapy or chemotherapy, surgery remains the most effective method for managing KIRC at present [3]. However, 20% to 30% of patients who undergo initial curative nephrectomy subsequently experience local or distant recurrence within five years [4]. While recurrence and metastasis of KIRC are associated with poor prognosis, promising new diagnostic tools and molecular-targeting agents have been developed over the past few decades [5]. KIRC is diagnosed primarily based on radiological examination and, when necessary, renal biopsy, but it is difficult to meet clinical requirements and achieve early detection with only these methods [6]. Novel effective biomarkers for early screening and diagnosis are therefore needed to improve therapeutic efficacy and patient quality of life.

While regulation of gene expression plays a crucial role in various biological processes, the impact of post-transcriptional gene regulation has attracted considerable attention recently [7]. For example, RNA-binding proteins (RBPs) regulate gene expression post-transcriptionally primarily by binding to RNA and forming ribonucleoprotein complexes [8]. RBPs are expressed in almost all cells, and more than 1,500 RBP genes accounting for about 7.5% of all protein-coding genes have been identified by high-throughput screening of the human genome [9]. Several possible functions have been proposed for RBPs, including RNA splicing, processing, transport, stability, localization, and degradation [10]. In addition, alterations in RBPs are strongly implicated in several human diseases. However, the precise roles RBPs play in tumor development and progression remain largely unknown.

In recent years, an increasing number of studies indicate that the expression of many RBPs is not only dysregulated in tumors compared to corresponding normal tissues, but is also closely related to tumor development and patient prognosis [11–13]. For example, MSI2 influenced breast cancer cell growth by binding to specific sites in ESR1 RNA and by increasing ESR1 protein stability [14]. Functional genomics analyses of RBPs identified the splicing regulator SNRPB as an oncogenic candidate in glioblastoma [15]. Yan et al. reported that KHSRP promoted tumor growth and metastasis in non-small cell lung cancer. Knockdown of KHSRP significantly reduced lung cancer cell proliferation, migration, and invasion in vitro and in vivo, and survival analysis showed that patients with high KHSRP expression levels had poor prognoses [16]. Although RBPs that may act as key regulators of tumor progression have been identified, only a small fraction of all human RBPs have been investigated in depth thus far. A systematic and comprehensive analysis of RBPs would not only provide new insights into the molecular mechanisms of tumor progression, but might also help identify innovative drug targets.

In this study, we identified multiple differentially expressed RBPs associated with KIRC using high-throughput bioinformatics analysis of data obtained from The Cancer Genome Atlas (TCGA). Subsequently, we evaluated relationships between expression profiles of differentially expressed RBPs and KIRC patient prognosis and constructed a risk model that served as an independent factor for predicting KIRC prognosis. Based on risk scores calculated using the model, we then established a nomogram for use as quantitative tool that could help physicians predict clinical outcomes and provide personalized treatments. Our results demonstrate that this risk model may serve as a promising prognostic indicator in KIRC patients.

## RESULTS

### Identification of differentially expressed RBPs in KIRC patients

**Figure 1:**
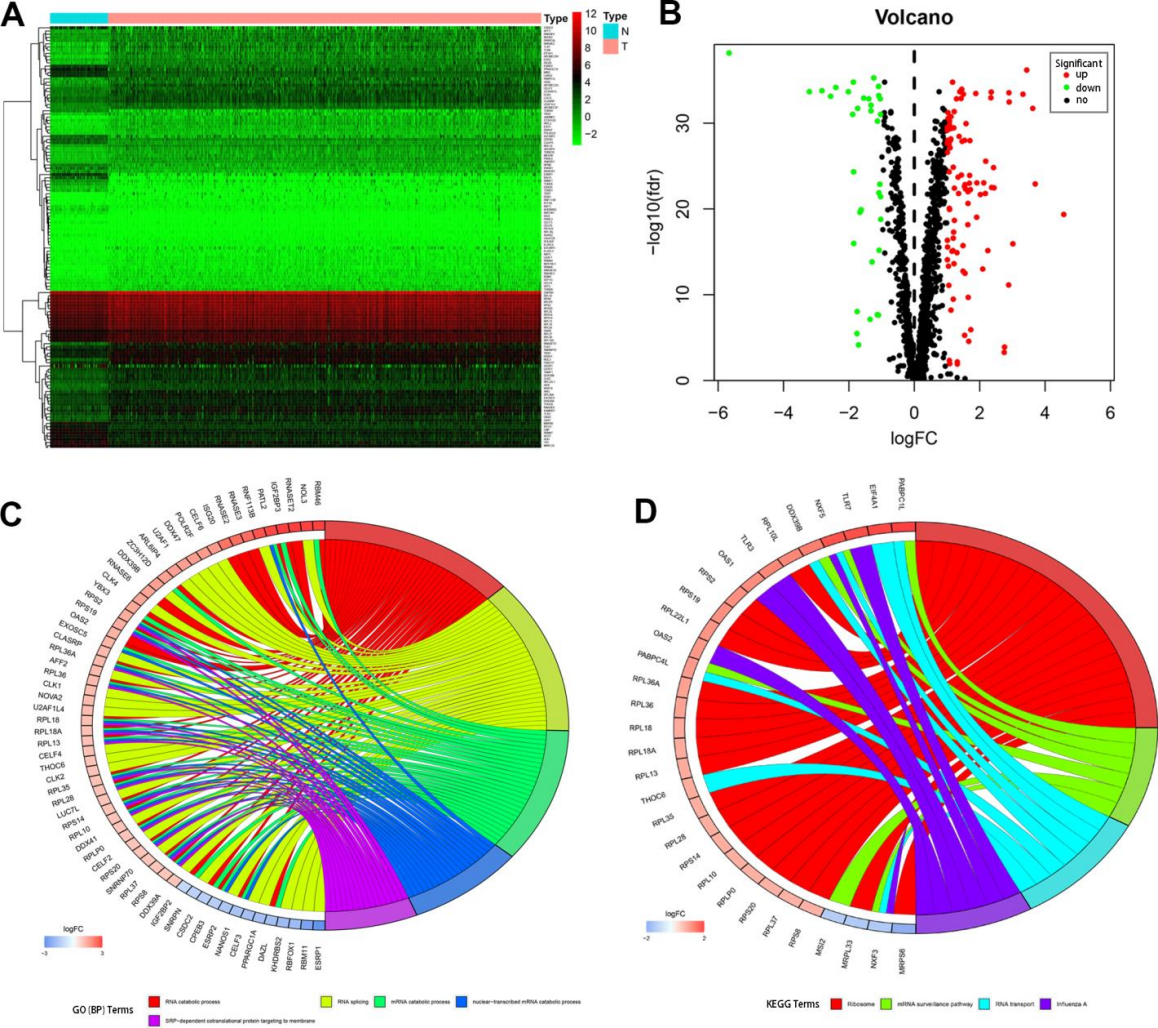
**Differentially expressed RBPs in kidney renal clear cell carcinoma and functional enrichments.** (**A**) Heatmaps of the differently expressed RBPs. Red and blue colors indicate higher and lower gene expression values, respectively. T indicates tumor tissues and N indicates non-tumor tissues. (**B**) Volcano plot of the differentially expressed RBPs. Red and blue colors indicate upregulated and downregulated RBPs, respectively. (**C**) Circos plot demonstrating relationships between selected GO-BP terms and associated RBPs. (**D**) Circos plot demonstrating relationships between selected KEGG terms and associated RBPs. Symbols for differentially expressed RBPs are shown on the left side of the graph. RBPs are ordered based on their logFC values. Relationships between RBPs and terms are indicated by colored connecting lines.

RNA-seq and clinical data from 539 tumor and 72 normal samples were downloaded from TCGA database. A total of 1542 RBPs were included in the present study. Of these, 133 RBPs with |log2FC| > 1.0 and FDR < 0.05 were considered differentially expressed genes; 98 were upregulated and 35 were downregulated. A heatmap and volcano plot showing expression distributions of these differently expressed RBPs are shown in Figure 1A, 1B.

### GO and KEGG pathway enrichment analysis of differentially expressed RBPs

To comprehensively understand the potential functions and molecular mechanisms of these RBPs, we carried out GO and KEGG pathway analysis both for all of the differentially expressed RBPs together and after separating them into upregulated and downregulated RBP groups. Regarding biological processes (BP), total differentially expressed RBPs were mostly enriched in RNA catabolic process, RNA splicing, mRNA catabolic process, nuclear-transcribed mRNA catabolic process, and SRP-dependent co-translational protein targeting to the membrane (Figure 1C). In addition, upregulated differentially expressed RBPs were significantly enriched in RNA catabolic process and both SRP−dependent and general co-translational protein targeting to the membrane, while downregulated RBPs were mostly enriched in regulation of mRNA metabolic process, regulation of RNA splicing, and RNA splicing (Table 1). Regarding cellular components (CC), the upregulated differentially expressed RBPs were significantly enriched in cytosolic ribosome, ribosomal subunit, and cytosolic large ribosomal subunit, while the downregulated RBPs were notably enriched in mitochondrial matrix, cytoplasmic ribonucleoprotein granule, and ribonucleoprotein granule (Table 1). In the molecular function (MF) category, the upregulated differentially expressed RBPs were associated with catalytic activity acting on RNA, structural constituent of ribosome, and nuclease activity, while the downregulated RBPs were enriched in mRNA 3'−UTR binding, translation regulator activity, and poly(U) RNA binding (Table 1).

**Table 1:** GO and KEGG enrichment analysis of aberrantly expressed RBPs.

	**Term**	***P* value**
**Up-regulated RBPs**		
Biological processes	RNA catabolic process	4.21E-24
	cotranslational protein targeting to membrane	3.07E-18
	SRP−dependent cotranslational protein targeting to membrane	1.71E-18
Cellular component	cytosolic ribosome	2.28E-21
	ribosomal subunit	2.35E-17
	cytosolic large ribosomal subunit	1.27E-16
Molecular function	catalytic activity acting on RNA	6.64E-16
	structural constituent of ribosome	3.87E-16
	nuclease activity	7.75E-10
KEGG pathway	ribosome	4.39E-16
	mRNA surveillance pathway	< 0.05
	influenza A	< 0.05
**Down-regulated RBPs**		
Biological processes	regulation of mRNA metabolic process	3.42E-09
	regulation of RNA splicing	1.21E-07
	RNA splicing	1.19E-06
Cellular component	mitochondrial matrix	< 0.01
	cytoplasmic ribonucleoprotein granule	< 0.001
	ribonucleoprotein granule	< 0.001
Molecular function	mRNA 3'−UTR binding	6.82E-07
	translation regulator activity	< 0.001
	poly(U) RNA binding	2.32E-05

KEGG pathway enrichment analysis revealed that all differentially expressed RBPs were significantly associated with ribosome, mRNA surveillance pathway, RNA transport, and influenza A (Figure 1D). More importantly, although upregulated differentially expressed RBPs were also mainly enriched in ribosome, RNA transport, and influenza A (Table 1), no significant enrichments were identified for downregulated RBPs.

### PPI network construction and key module analysis

**Figure 2:**
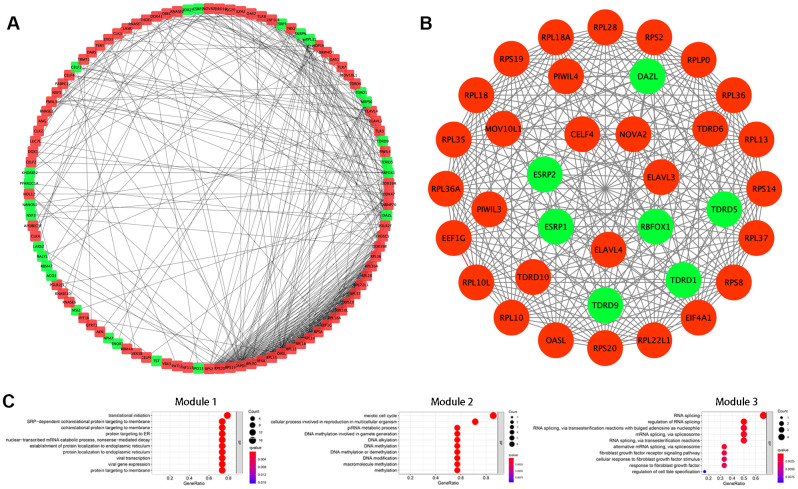
**Protein-protein interaction (PPI) network construction and modules analysis.** (**A**) PPI network of differentially expressed RBPs. (**B**) The top three significant modules from the PPI network. Red circles indicate upregulated RBPs and green circles indicate downregulated RBPs. (**C**) GO-BP enrichment analysis of the top three significant modules.

Next, we further explored the effects of the 133 differentially expressed RBPs in KIRC using a PPI network. Interaction relationship data for the RBPs were downloaded from the STRING tool and imported into Cytoscape for visualization. The PPI network consisted of 108 nodes and 463 edges (Figure 2A). We then analyzed the co-expression network further to identify possible key modules using the MODE plugin and identified the three most significant modules (Figure 2B). GO-BP enrichment analysis revealed that the RBPs from module 1 were highly associated with SRP-dependent co-translational protein targeting to the membrane, co-translational protein targeting to the membrane, and protein targeting to the ER. The RBPs in module 2 were mainly enriched in DNA methylation involved in gamete generation, DNA alkylation, and DNA methylation. The RBPs in module 3 were mainly enriched in regulation of RNA splicing, RNA splicing, and alternative mRNA splicing via the spliceosome (Figure 2C).

### Identification of prognostic RBPs and construction of the prognostic risk model

**Figure 3:**
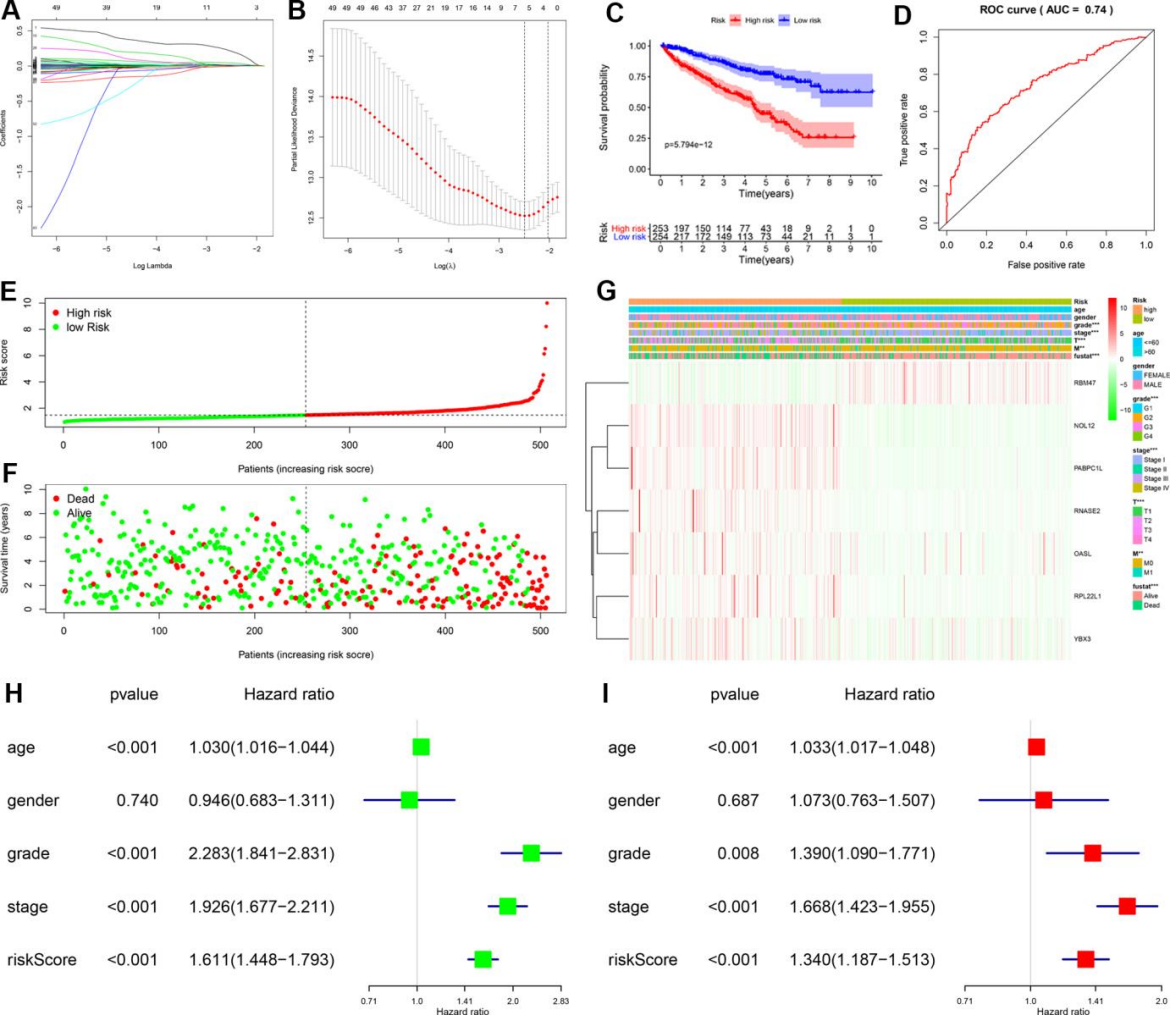
**Construction and validation of prognostic risk score for KIRC patients.** (**A**, **B**) Selection process for target RBPs using LASSO Cox regression analysis. (**C**) Kaplan-Meier curve showing that OS is significantly shorter for patients in the high-risk group than those in the low-risk subgroup. (**D**) ROC curve analysis showing the veracity and reliability of the prognostic model. (**E**) Risk score distributions. (**F**) Scatter plot showing the distribution of survival status and survival time. (**G**) Grade, AJCC stage, T stage, M stage, and survival status differ significantly between the high- and low-risk groups. Univariate (**H**) and multivariate (**I**) Cox regression analysis of associations between clinical parameters (including risk score) and overall survival of KIPC patients.

The PPI network was used to identify 108 key differentially expressed RBPs. A univariate Cox regression analysis of their prognostic value identified 56 prognosis-related candidate RBPs (*P* < 0.05). We then performed LASSO regression analysis to identify RBPs with the highest potential prognostic significance. Ultimately, 7 target RBPs were retained and used to construct a predictive model (Figure 3A, 3B). Among these 7 RBPs, NOL12, PABPC1L, RNASE2, RPL22L1, OASL, and YBX3 were risk genes; only RBM47 was not a risk gene.

We then used expression levels of the 7 target RBPs and the regression coefficients determined above to calculate a risk score for each patient. Risk scores were calculated using the following equation: Risk score = (0.2072 * NOL12) + (0.0103 * PABPC1L) + (0.0433 * RNASE2) + (0.0121 * RPL22L1) + (-0.0060 * RBM47) + (0.0032 * OASL) + (0.0003 * YBX3). Data from KIRC patients in the TCGA dataset were dichotomized into high- and low-risk groups using the median risk score as a cutoff. As shown in Figure 3C, OS was lower in the high-risk group than in the low-risk group. Furthermore, the AUC of the ROC curve generated to evaluate the prognostic ability of the model was 0.74, suggesting that the seven-RBP model had moderate diagnostic performance (Figure 3D). Next, we ranked patients according to their risk scores (Figure 3E) and analyzed the survival status of each patient on a dot plot. The results revealed that survival rates and times were higher for patients in the low-risk group than for those in the high-risk group (Figure 3F). Expression levels of the seven target RBPs in the high- and low-risk groups are shown in Figure 3G. Expression of NOL12, PABPC1L, RNASE2, RPL22L1, OASL, and YBX3 was significantly higher, while RBM47 expression was significantly lower, in the high-risk group than in the low-risk group. Tumor grade, AJCC stage, T stage, M stage, and survival status also differed significantly between the high- and low-risk groups (all *P* < 0.001).

### Independent prognostic analysis of the risk model

To determine whether the risk score was an independent prognostic factor, we performed univariate and multivariate Cox regression analyses of TCGA data. The univariate analysis showed that age, grade, AJCC stage, and risk score were significantly correlated with OS in KIRC patients (Figure 3H). Furthermore, multivariate analysis indicated that age, grade, AJCC stage, and risk score were all independent prognostic factors for OS (Figure 3I). Collectively, these analyses demonstrated that the risk score based on seven RBPs might serve as an independent prognostic factor for KIRC patient survival.

### Correlations between the prognostic model and clinical parameters

**Figure 4:**
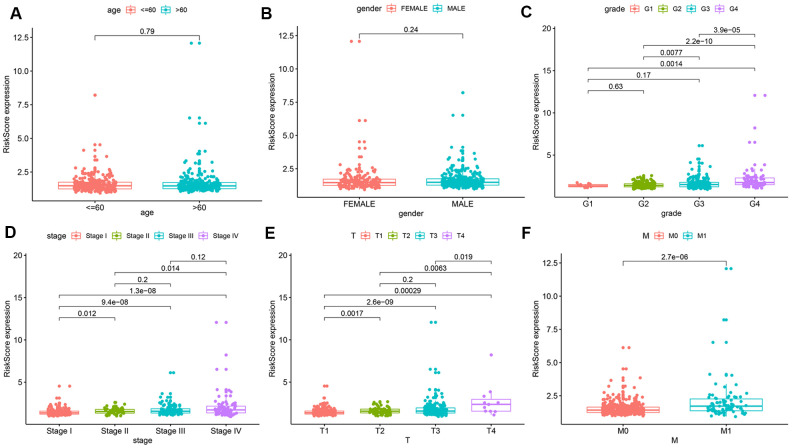
**Relationships between risk score and various clinical parameters.** Risk scores in cohorts stratified by age (**A**), gender (**B**), grade (**C**), AJCC stage (**D**), T stage (**E**), and M stage (**F**). Risk score is significantly associated with grade, AJCC stage, T stage, and M stage, but not with age or gender.

To further explore the prognostic value of the risk model, we analyzed relationships between the risk score and various clinical parameters (Figure 4). The results indicated that risk score was significantly associated with grade, AJCC stage, T stage, and M stage; the higher the risk score, the greater the probability of progression to advanced tumors. However, risk scores were not associated with age or gender.

**Figure 5:**
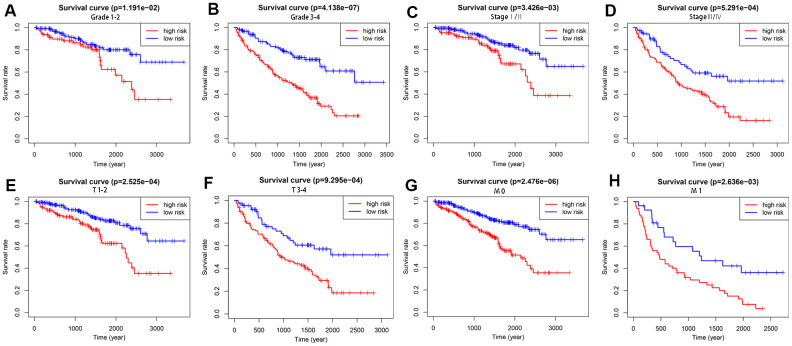
**Survival differences between high- and low-risk KIRC patients stratified by clinical factors.** Differences in overall survival in patients stratified by grade (**A**, **B**), AJCC stage (**C**, **D**), T stage (**E**, **F**), and M stage (**G**, **H**). Kaplan–Meier survival curves show that patients in the high-risk group have significantly poorer OS than those in the low-risk group in all subgroups.

To explore the wider applicability of this risk model, all KIRC patients were classified into several stratified cohorts according to different clinicopathological parameters. Kaplan–Meier survival curves showed that patients in the high-risk group had significantly poorer OS than those in the low-risk group in all subgroups (Figure 5).

Additionally, to better assess the functions of the seven RBPs included in the model in disease progression, we also analyzed correlations between the expression of each RBP and clinicopathological features (Table 2). The results indicated that, as NOL12, PABPC1L, RNASE2, RPL22L1, and OASL expression increased, tumor grade, AJCC stage, T stage, and M stage in KIRC patients increased. In addition, as YBX3 expression, which was higher in males than in females, increased, AJCC stage, T stage, and M stage in KIRC patients also increased. In contrast, as RBM47 expression, which was higher in females than in males, increased, tumor grade, AJCC stage, and T stage in KIRC patients decreased. Consistent with these findings, NOL12, PABPC1L, RNASE2, RPL22L1, OASL, and YBX3 contributed to tumor progression, while RBM47 exerted protective effects against disease progression.

**Table 2:** Correlation analysis between seven RBPs and clinical variables for KIRP.

**Variables**	**Age (≤65, >65) t(*p*)**	**Gender (Female, Male) t(*p*)**	**Grade (1/2, ¾) t(*p*)**	**AJCC Stage (I/II, III/IV) t(*p*)**	**T Stage (1/2, ¾) t(*p*)**	**M Stage (1/2, ¾) t(*p*)**
NOL12	-0.008	1.122	-2.468*	-3.748***	-3.519***	-2.076*
PABPC1L	-0.845	1.336	-3.875***	-3.718***	-3.407***	-2.388*
RNASE2	-0.627	-1.463	-4.234***	-3.677***	-3.441***	-2.407*
RPL22L1	-0.380	-1.060	-4.676***	-4.929***	-4.034***	-3.701***
RBM47	-0.137	2.545*	3.009**	3.944***	3.974***	1.748
OASL	-1.967	-0.732	-5.065***	-5.583***	-4.437***	-4.451***
YBX3	-1.046	-2.548*	-1.149	-4.628***	-4.323***	-2.362*

t: t value from Student’s *t* test; *p*: *p*-value from Student’s *t* test.

* *p*< 0.05; ** *p*< 0.01; *** *p*< 0.001.

### Building a predictive, risk score-based nomogram

**Figure 6:**
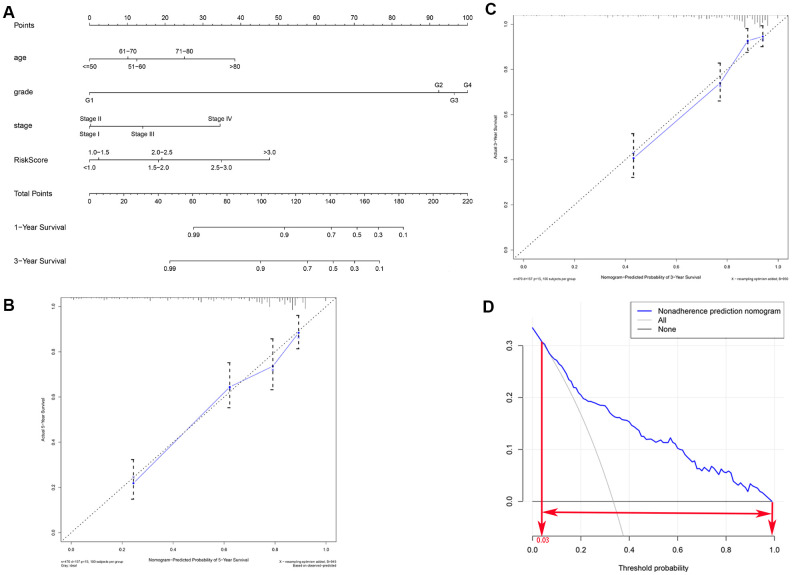
**Clinical prognostic nomogram for predicting prognosis in TCGA KIRC cohorts.** (**A**) The clinical nomogram developed to predict 3- and 5-year survival by incorporating four independent prognostic indicators, including risk score. Calibration curves showing nomogram predictions for 3-year (**B**) and 5-year (**C**) survival. (**D**) Decision curve analysis was used to estimate clinical usefulness and net benefit of the predictive nomogram.

In order to develop a quantitative method for predicting prognosis in KIRC patients, we constructed a nomogram that integrated risk score and the independent predictors identified above (age, grade, and AJCC stage). Each variable was assigned a score; the scores of the four variables were then added, and a vertical line was drawn from the total score to the nomogram points scale to determine estimated 3-year and 5-year survival rates (Figure 6A). The C-index value of the prediction nomogram was 0.792 for the KIRC cohort, indicating that it had good discrimination capability. The calibration curves indicated that the nomogram predictions were very consistent with actual observations for 3- and 5-year OS in the TCGA-KIRC cohort, suggesting that the nomogram was reliable (Figure 6B, 6C). Additionally, DCA was used to evaluate the clinical efficiency of the predictive nomogram. The results showed that the nomogram could improve prognosis predictions for patients with a threshold probability of > 3% (Figure 6D).

### Gene set enrichment analysis (GSEA)

**Figure 7:**
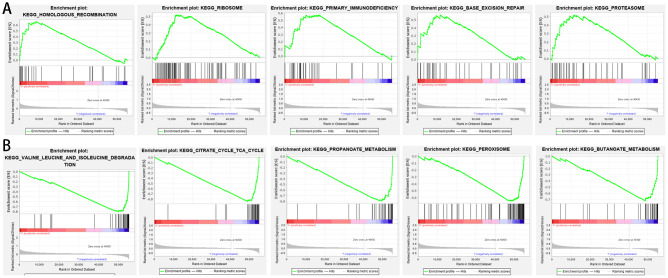
**Gene set enrichment analysis between high- and low-risk groups based on the prognostic risk model.** (**A**) High-risk group. (**B**) Low-risk group.

GSEA was performed to further understand functional differences between the high- and low-risk groups identified by the prognostic model. High risk scores were significantly associated with homologous recombination (NES=1.878, *P*=0.000), ribosome (NES=1.763, *P*=0.000), primary immunodeficiency (NES=1.714, *P*=0.001), base excision repair (NES=1.669, *P*=0.004), and proteasome (NES=1.628, *P*=0.005) (Figure 7A). Meanwhile, low risk scores were closely associated with valine leucine and isoleucine degradation (NES=-3.3150, *P*=0.000), citrate cycle (NES=-3.262, *P*=0.000), propanoate metabolism (NES=-3.189, *P*=0.000), peroxisome (NES=-2.997, *P*=0.000), and butanoate metabolism (NES=-2.948, *P*=0.000) (Figure 7B). Interestingly, several metabolic processes were associated with the low-risk group; these pathways merit further investigation.

### The prognostic value of target RBPs

**Figure 8:**
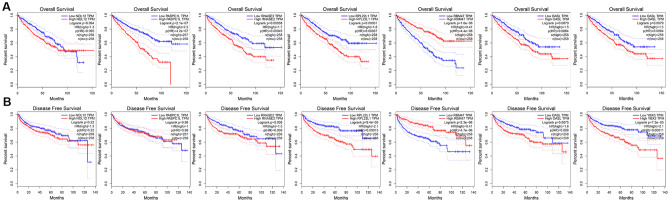
**Univariate survival analysis of the target RBPs using Kaplan-Meier curves.** (**A**) Relationships between RBPs and OS. (**B**) Relationships between RBPs and DFS.

To further explore the prognostic value of the target RBPs in KIRC, survival plots were used to evaluate relationships between RBPs and OS in the GEPIA database. Log-rank tests indicated that higher PABPC1L, RNASE2, RPL22L1, YBX3, and OSAL expression was associated with shorter OS. In this analysis, higher RBM47 expression was also associated with shorter OS. However, no significant differences in OS were observed between patients with high and low NOL12 expression (Figure 8A). Similar trends were also observed between RBPs and disease-free survival (DFS) in the GEPIA database. Log-rank tests suggested that higher RNASE2, RPL22L1, YBX3, and OSAL expression was associated with shorter DFS. Again, higher RBM47 expression was also associated with shorter DFS. No significant differences in DFS were observed between patients with high and low NOL12 and PABPC1L expression (Figure 8B).

### Validation of target RBP expression levels

**Figure 9:**
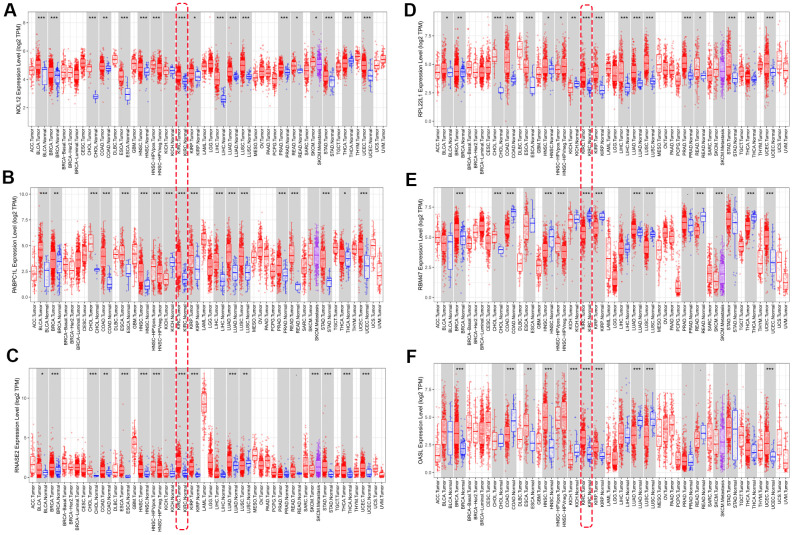
**Verification of target RBP expression in KIRC and normal tissues using the TIMER database.** (**A**) NOL12, (**B**) PABPC1L, (**C**) RNASE2, (**D**) RPL22L1, (**E**) OSAL, and (**F**) RBM47.

We used ‘DiffExp module’ from the TIMER database to examine the expression of the seven target RBPs in multiple tumors, including KIRC. Consistent with the above results, NOL12, PABPC1L, RNASE2, RPL22L1, and OSAL expression were significantly increased, while RBM47 expression was significantly decreased (Figure 9). YBX3 expression data was not available in the TIMER database, so it was excluded from this analysis. Interestingly, aberrant RBP expression was common in various malignancies and exhibited some degree of tissue specificity. For example, OASL was overexpressed in breast cancer and esophageal cancer, but reduced in colon cancer and lung cancer.

**Figure 10:**
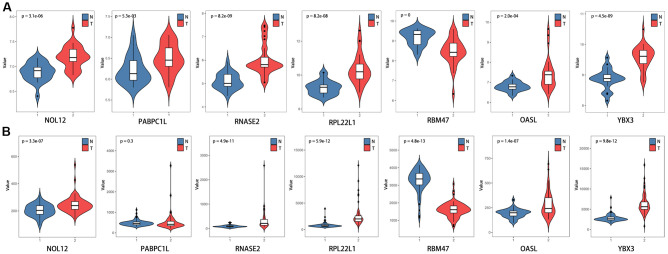
**Verification of target RBP expression in KIRC and normal tissues using the GEO database.** (**A**) GSE36895, (**B**) GSE53757.

The differentially expressed RBPs were further verified using the GSE36895 (Figure 10A) and GSE53757 (Figure 10B) datasets, which generally yielded results consistent with our previous findings. However, PABPC1L levels did not differ between tumor and normal tissues in the GSE53757 dataset, perhaps due to differences in study populations and measurement scales.

**Figure 11:**
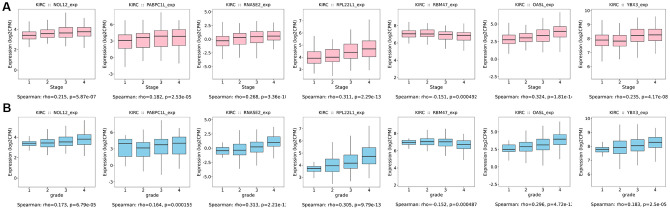
**Box diagrams of target RBPs across different grades and AJCC stages in the TISIDB online database.** (**A**) Box diagram showing the expression level of target RBPs in different tumor grades. (**B**) Box diagram showing the expression level of target RBPs in different AJCC stages.

Additionally, we further verified correlations between target RBPs and KIRC tumor grade and AJCC stage using the TISIDB online database. The results indicated that the seven target RBPs were not only closely associated with tumor grade, but also with AJCC stage (Figure 11). NOL12, PABPC1L, RNASE2, RPL22L1, OASL, and YBX3 expression were significantly positively correlated with tumor grade and AJCC stage, while RBM47 expression was negatively correlated with tumor grade and AJCC stage.

## DISCUSSION

Despite recent progress in various treatment strategies, prognoses for many advanced stage KIRC patients remain dismal [17], and novel biomarkers are needed to improve early screening and to better monitor tumor progression. Many reports have confirmed that dysregulated RBP expression is closely related to development and progression of multiple tumor types [18, 19]. However, only a relatively small number of RBPs have been studied extensively enough to establish direct links to cancer development and progression [20].

In this study, we examined RBP expression profiles using data from the TCGA database and identified RBPs that were differentially expressed between KIRC and non-tumor tissues. We then systematically analyzed the potential biological functions of these RBPs and established PPIs. Using Lasso regression and Cox survival analyses, we constructed a risk model based on seven prognostic RBPs: NOL12, PABPC1L, RNASE2, RPL22L1, RBM47, OASL, and YBX3. Moreover, GSEA was used to further understand the functional differences associated with RBPs differentially expressed between high- and low-risk groups based on the prognostic model. Subsequently, we built a predictive nomogram to improve the accuracy of 3- and 5-year OS predictions by incorporating the risk score and several clinical parameters. Together, our results demonstrated that the risk model accurately predicted prognoses of KIRC patients and might help improve diagnosis and clinical treatments.

FGO and KEGG pathway analysis were performed to identify functional pathways in which RBPs were enriched. The most commonly enriched biological processes were RNA catabolic process, nuclear-transcribed mRNA catabolic process, mRNA metabolic process, and RNA splicing, all of which are known to affect development and progression of various diseases [21–23]. In addition, cytosolic ribosomes and the mitochondrial matrix were the most common locations associated with functional enrichment. Ribosomes are responsible for protein synthesis, and different experimental models indicated ribosomal proteins may initiate cancer development by regulating mRNA translation and p53 activity [24]. Alterations in mitochondrial functions are also required to maintain tumor viability [25]. Regarding molecular functions, the differentially expressed RBPs were significantly enriched in catalytic activity acting on RNA, structural constituent of ribosome, nuclease activity, mRNA 3'−UTR binding, translation regulator activity, and poly(U) RNA binding. In addition, KEGG pathway analysis indicated that these aberrantly expressed RBPs might affect the development and progression of KIRC by regulating ribosomes, the mRNA surveillance pathway, and RNA transport. These results suggest that RBPs can affect tumor cell survival and growth by regulating a variety of biological processes.

Next, we identified a module that included 108 key RBPs by constructing a PPI network based on all of the differently expressed RBPs. Univariate Cox regression analysis indicated that 56 of the RBPs were significantly related to OS in KIRC patients. Finally, LASSO regression analysis was performed to identify the final seven target RBPs that were used to construct a prognostic risk model. Patients in the high-risk group had shorter OS than those in the low-risk group; this difference was also observed in other cohorts in which patients were stratified differently. Furthermore, as risk score increased, the probability of tumor progression to advanced stages also increased. These results suggested that our risk model served as an independent and accurate predictor of KIRC patient prognosis. A nomogram that incorporated the risk score and clinical features, specifically age, grade, and AJCC stage, was then built to estimate 3-year and 5-year survival rates of KIRC patients. C-index and calibration plots indicated that the nomogram was reliable had good discrimination capability. Moreover, GSEA revealed that high risk scores were associated with differences in homologous recombination and ribosome. Interestingly, patients in the low-risk group were significantly enriched in metabolism pathways, such as valine leucine and isoleucine degradation, propanoate metabolism, and butanoate metabolism. This indicates that low-risk patients might benefit more from metabolic therapy; future experiments and bioinformatics analyses should examine this possibility.

Roles for most of the RBPs in our risk model have been reported previously in different tumors. NOL12 is a multifunctional RNA binding protein at the nexus of RNA and DNA metabolism, and NOL12 inhibition can contribute to stabilization and activation of p53 in an RPL11-dependent manner, thereby preventing the occurrence of cancer [26, 27]. Zhang et al. comprehensively analyzed RBP expression profiles and found that PABPC1L might promote colon cancer progression by regulating mRNA splicing [28]. Another study showed that PABPC1L depletion inhibited proliferation and migration by blocking the AKT pathway in human colorectal cancer cells [29]. RNASE2, a member of the mammalian ribonuclease gene family, might play an important role in immune response modulation and TLR2 activation [30]. RNASE2 is overexpressed in some cancers, including acute lymphoblastic leukemia and colorectal cancer [31, 32]. RPL22L1 is critical in maintaining an aggressive phenotype in ovarian cancer and in triggering cell metastasis by inducing epithelial-to-mesenchymal transition (EMT) [33]. Previous research indicated that OASL may be crucial for maintaining lung cancer cell susceptibility to Actinidia chinensis Planch root extract and might be associated with the development of drug resistance [34]. Previous evidence indicates that YBX3 upregulation promotes gastric cancer pathogenesis by increasing cell invasion and tumor chemoresistance [35]. As a tumor suppressor gene, RBM47 inhibits tumor cell growth through the inhibition of Nrf2 activity in lung adenocarcinoma, and RBM47 knockdown enhanced tumor formation and metastasis in a xenograft mouse model [36]. Consistent with our present findings, these results demonstrate that increased expression of these RBPs, except for RBM47, is significantly associated with poorer prognosis in KIRC patients. In addition, the seven target RBPs were validated using sequencing databases, including TIMER and GEPIA, and those results further confirmed our findings.

The major innovation of our study centers on systematic analysis of a large patient cohort from the TCGA database, which allowed us to construct a strong prognostic model based on seven RBPs that might have significant value in clinical applications. To the best of our knowledge, the analysis of the correlation between KIRC and RBPs has not yet been investigated and the results from the present study may therefore be considered as relevant for prognosis in KIRC. Furthermore, our prognostic model also predicted survival accurately when patients were stratified into different cohorts based on other disease characteristics and might therefore help physicians make more precise individualized survival predictions. However, some limitations of our study should be noted when interpreting the results. First, some important clinical characteristics of KIRC patients, such as living environment, smoking history, and family history, were not available in the TCGA database; inclusion of these factors might increase the efficacy and reliability of the model. Second, the inherent limitations of a retrospective study design apply here, and prospective studies are needed to validate the results. Finally, the results of the bioinformatics analyses presented here should be verified with in vitro and in vivo experiments.

In conclusion, we performed a comprehensive bioinformatics analysis to investigate the prognostic value of aberrantly expressed RBPs in KIRC patients based on data from TCGA. We built a prognosis risk model containing seven target RBPs that could independently and accurately predict the survival of KIRC patients. Expression of these RBPs may be closely correlated with clinicopathological characteristics of KIRC. A prognostic nomogram that generates individualized survival predictions was also constructed by combining the prognosis model-based risk score and clinical parameters; this might be particularly helpful in guiding clinical decisions. This model might therefore help identify novel prognostic markers and potential therapeutic targets for KIRC patients.

## MATERIALS AND METHODS

### Data acquisition and identification of differentially expressed RBPs

RNA sequencing transcriptome data for 539 KIRC samples and 72 normal samples with corresponding clinical information were downloaded from the TCGA (The Cancer Genome Atlas, https://cancergenome.nih.gov/) database. Patients with incomplete data or survival times of less than 30 days were excluded. The list of RNA binding proteins (RBPs) used in this study was retrieved from a recent study by Gerstberger et al. [9]. Differential expression analysis was performed on raw counts using the Limma package (http://www.bioconductor.org/packages/release/bioc/html/limma.html), and genes with an average count value greater than 0 were examined in subsequent analysis. We used the Limma package to identify RBPs that were differentially expressed between cancer samples and normal control samples. A false discovery rate (FDR) < 0.05 and |log2 fold change (FC)| > 1.0 were chosen as threshold values. Ethical approval and informed consent requirements were waived because the data were obtained from public databases.

### Functional enrichment of differentially expressed RBPs

Gene ontology (GO) enrichment analysis was carried out to comprehensively explore the biological functions of the differentially expressed RBPs. GO terms were divided into three broad categories: biological process (BP), cellular component (CC), and molecular function (MF). Biological pathways associated with the differentially expressed RBPs were analyzed using the Kyoto Encyclopedia of Genes and Genomes database (KEGG). All enrichment analyses were conducted in R software (version 3.6.2) using the ‘ClusterProfiler,’ ‘ggplot2,’and ‘GOplot’ functions to visualize the results. A *P*-value of less than 0.05 was considered significant.

### Protein–protein interaction (PPI) network and key modules analysis

The STRING database (http://string-db.org) was used to analyze protein-protein interactions (PPI) among the differentially expressed RBPs. Analyzing functional interactions between proteins can provide new insights into their functions and contribute to the discovery of functional connections between proteins at the genome-wide level. Data obtained from the STRING database were imported into Cytoscape (http://cytoscape.org/) software for network visualization. In addition, Molecular Complex Detection (MCODE), a plugin for Cytoscape, was used to screen significant functional modules of the PPI network using score and number of nodes > 2 as thresholds.

### Construction and evaluation of the prognostic model

Univariate Cox analysis was performed to evaluate associations between expression levels of key RBPs and overall survival (OS) for the TCGA KIRC cohort using the R ‘survival’ package. RBPs with *P*-value less than 0.05 in the univariate analysis were considered significantly correlated with OS. Hazard risk (HR) was calculated to identify risk-increasing genes (HR > 1) and protective genes (HR < 1). Least absolute shrinkage and selection operator (LASSO) analysis, a robust model-building method that prevents over-fitting, was used to further narrow the range of genes and obtain an optimal predictive model. Surviving target genes were used to construct the prognostic model. A risk score was calculated for each patient based on a linear combination of each target gene expression level weighted by the regression coefficient derived from LASSO analysis.

All KIRC patients were dichotomized into high-risk and low-risk groups based on the median risk score. A survival curve was plotted to determine survival rate using the Kaplan-Meier method, and differences in OS between high- and low-risk groups were compared using the log-rank test. Generally, patients with higher risk scores were expected to have a shorter OS. Additionally, the receiver operating characteristic (ROC) curve and area under the ROC curve (AUC value) were calculated to evaluate the diagnostic performance of the prognostic model. Distributions of clinical variables between high- and low-risk groups were compared using a chi-squared test.

Univariate Cox regression survival analysis was used to evaluate the prognostic value of risk score and various clinical parameters including age, gender, tumor grade, and AJCC stage. To further validate whether the risk score could serve as an independent prognostic factor for KIRC patients, multivariate Cox regression analysis was then performed. A Kruskal-Wallis test was applied to compare risk score values of different subgroups in order to better understand the prognostic value of the constructed model. We also analyzed correlations between expression levels of the individual RBPs in the model and the clinicopathological features to better assess the functions of the seven RBPs in disease progression.

### Development of a predictive nomogram

Nomograms are important in the modern medical decision-making process because they can help predict the probability of a clinical event by integrating diverse prognostic and determinant variables [37]. Each independent prognostic factor identified above was therefore included in the predictive nomogram developed here. Concordance index (C-index) and calibration curves were then used to evaluate its discriminatory capacity and predictive accuracy, respectively. Decision curve analysis (DCA) was conducted to estimate the clinical usefulness and net benefit of the predictive nomogram.

### Gene set enrichment analysis (GSEA)

To explore KEGG pathways associated with the prognostic model, Gene Set Enrichment Analysis (GSEA) was used to identify enriched terms in the high- and low-risk groups of the TCGA KIRC cohort. GSEA was performed on these pre-ranked genes using GSEA software (http://www.broadinstitute.org/gsea) using 1,000 permutations and gene set sizes between 15 and 500. A nominal *P* value < 0.05 and FDR value < 0.25 were considered statistically significant.

### Validation of target RBPs

A good model would not only predict oncologic outcome for KIRC patients, but also improve diagnostic accuracy in differentiating benign from malignant masses. Differences in expression levels of the prognostic genes in the model between tumor and normal tissues were therefore further validated at the translational level using the Tumor Immune Estimation Resource (TIMER, https://cistrome.shinyapps.io/timer/) database. The identified differences in RBP expression were then verified in two independent cohorts (GSE36895 and GSE53757) downloaded from Gene Expression Omnibus (GEO) database (https://www.ncbi.nlm.nih.gov/geo/).

Overall survival analysis and disease-free survival analysis were performed to explore the potential prognostic value of each target RBP in our model using the Gene Expression Profiling Interactive Analysis (GEPIA, http://gepia.cancer-pku.cn/) online tool. In addition, the tumor-immune system interactions (TISIDB, http://cis.hku.hk/TISIDB/index.php) online database was used to verify correlations between target RBP expression and grade or AJCC stage.
